# FDPS promotes glioma growth and macrophage recruitment by regulating CCL20 via Wnt/β‐catenin signalling pathway

**DOI:** 10.1111/jcmm.15542

**Published:** 2020-06-28

**Authors:** Zhuo Chen, Guangyong Chen, Hang Zhao

**Affiliations:** ^1^ Neurosurgery Department The Third Hospital of Jilin University Changchun China

**Keywords:** CCL20, FDPS, glioma, TAMs, Wnt/β‐catenin

## Abstract

Glioma is one of the most lethal tumours and common malignant in the central nervous system (CNS), which exhibits diffuse invasion and aggressive growth. Several studies have reported the association of FDPS to tumour development and progression. However, the role of FDPS in progression of glioma and macrophage recruitment is not well‐elucidated. In the current study, a remarkable enhancement in FDPS level was observed in glioma tissues and associated with poor prognosis, contributed to tumour growth. FDPS was correlated with macrophage infiltration in glioma and pharmacological deletion of macrophages largely abrogated the oncogenic functions of FDPS in glioma. Mechanistically, FDPS activated Wnt/β‐catenin signalling pathway and ultimately facilitates macrophage infiltration by inducing CCL20 expression. In conclusion, overexpressed FDPS exhibits an immunomodulatory role in glioma. Therefore, targeting FDPS may be an effective therapeutic strategy for glioma.

## 1 INTRODUCTION

Glioma is one of the most malignant tumours in brain, which accounting for the majority of brain cancer‐related deaths. Gliomas include astrocytomas, oligodendrogliomas and glioblastoma multiforms (GBM), which were classified as grade I‐IV based on histological characteristics and genetic changes.^1^, ^2^ Although standard therapies include radiotherapy and concomitant, maximum safe surgical resection and maintained temozolomide (TMZ) chemotherapy, the prognosis of glioma patients stills poor and almost all patients inevitably relapse.^3^, ^4^ In particular, the median survival time of glioma patients is still about 1 year, and the 5‐year overall survival rate is less than 5%.^5^, ^6^ Therefore, it is particularly important to further explore the mechanisms underlying glioma development and to seek new approaches to treat glioma.

An enzyme of mevalonate pathway, farnesyl diphosphate synthase (FDPS) is expressed at high levels in glioma and several other cancers.^7^ Among the genes of mevalonate pathway, the recurrence and aggressiveness of glioma was seen to be closely related to FDPS.^8^ Furthermore, FDPS was found to be one of the highest differentially expressed genes when a global RNA sequencing analysis of glioma tissues was carried out, and correlated with progress, advancement and recurrence of glioma.^9^ Being a pleiotropic enzyme, FDPS catalyses the reaction between dimethylallyl pyrophosphate and isopentenyl pyrophosphate, and leads to the production of FDP (farnesyl diphosphate) and geranyl diphosphate.^10^ FDPS has a crucial part in protein prenylation and CBS (cholesterol biosynthesis).^11^ In the process of posttranslational modification, the farnesyl group is transferred from FDP to small GTPases including Ras, Rab, Rac, Rap1α and Rho proteins, and the reaction is catalysed by FTase (farnesyl transferase) enabling them in anchoring to membranes and signalling pathways of the cell.^12^ For several decades, mevalonate pathway enzymes (including FDPS) were examined for their role in cellular physiology, although, their role in various cancers including glioma remains elusive.^13^

Several characteristics of glioma, such as metastasis and tumour angiogenesis are modulated by TAMs (tumour‐associated macrophages).^14^ The two primary phenotypes exhibited by TAMs include M1 and M2, which normally exert contrasting outcomes on advancement of the tumour.^15^ The macrophages M1 have been long established to be activated macrophages, polarized by IFN‐γ (interferon‐γ) and LPS (lipopolysaccharide).^16^ They express IL‐1β (interleukin‐1β), IL‐12 and cytotoxic substances such as iNOS (inducible nitric oxide synthase).^17^ Contrarily, macrophages M2 are alternatively activated macrophages, polarized in the presence of IL‐4, IL‐10 or IL‐13.^18^ They express IL‐6 and IL‐10, and angiogenic factors like VEGF (vascular endothelial growth factor).^19^ Infiltration of M2 at high levels is related to poor prognosis in patients of glioma.^20^, ^21^ While the microenvironment of the tumour regulates differentiation of TAMs to M1 or M2 phenotypes, the polarization of macrophages is regulated by several microenvironment signals gained from tumour cells.^22^, ^23^

In the current study, we evaluated the dysregulated FDPS expressed by glioma cells and uncover its oncogenic functions in glioma. Our results indicated that FDPS is up‐regulated in glioma tissues compared with normal tissues and that FDPS overexpression promotes glioma proliferation and TAMs recruitment by regulating CCL20. Pathway analysis illustrated that Wnt/β‐catenin signalling pathway is involved in CCL20 induction and TAMs recruitment in glioma. Together, our results suggest that FDPS is a novel target for overcoming chemoresistance in glioma.

## 2 MATERIALS AND METHODS

### 2.1 Cell lines and regents

The human glioma cell lines U87, U251, H4, A172 and U118 cells were procured from ATCC (American Type Culture Collection, Manassas, VA, USA). Glioma cells maintained in RPMI‐1640 medium were then cultures in DMEM from Gibco BRL (Grand Island, NY, USA). NHAs (Normal human astrocytes) were procured from Lonza (Basel, Switzerland) and cultured as per supplied instructions. Each medium was augmented with FBS (foetal bovine serum, 10%). The cells were then further kept with 5% CO_2_ at 37°C.

### 2.2 Tissue specimens

Glioma fresh tissues and normal tissues adjacent to the tumours were collected from The Third Hospital of Jilin University from 2015 to 2017. Survival rate analysis was collected from 40 patients at The Third Hospital of Jilin University from 2013 to 2017. All included patients provided informed consent, and the Ethics Committee of The Third Hospital of Jilin University approved this study.

### 2.3 Immunohistochemistry

Immunohistochemistry was conducted as per a previous protocol.^24^ Briefly, slides were kept overnight for antibody incubation at 4°C and then assessed by two independent pathologists who were blinded for the study. For validation, the criteria for scoring was as previously described.^25^ The expression of FDPS was scored and categorized into four grades based on intensity of staining (0, 1+, 2+ and 3+) as well as the share of FDPS‐positive cells in percentage: 0 (0%‐10%), 1 (10%‐29%), 2 (30%‐59%) and 3 (>60%). Then, the scores of these two parameters, which ranged from 0 to 9, were multiplied to obtain a final staining score. To further analyse, weakly stained and negative FDPS cases were consolidated into one group and were contrasted with glioma cases that were moderately and strongly FDPS‐positive.

### 2.4 Assays for in vitro migration, invasion and proliferation

The determination of cell proliferation was done by colony formation assay, Cell‐Light EdU (EdU DNA Cell Proliferation) assay from Ribobio (Guangzhou, China), MTS assay from Promega (Madison, WI, USA). The cell invasion and migration were assessed through transwell and wound healing assays and were carried out as described.

### 2.5 The lentivirus constructs

The constructs of shRNA against FDPS (CCAGCAGTGTTCTTGCAATAT) and scrambled sequences (CCTAAGGTTAAGTCGCCCTCG) were procured from GenePharma, Shanghai (China). Three‐plasmid system (pPACKH1‐REV, pPACKH1‐GAG and pVSV‐G) were used to produce lentiviral particles which were packaged in 293T cells using Lipofectamine 3000 from Invitrogen (Carlsbad, CA, USA) as per instructions of the manufacturer. Infection of cells with recombinant lentivirus‐transducing units (1 × 10^6^) was done along with 6 μg/mL polybrene (Sigma‐Aldrich, St. Louis, MO, USA). The uninfected cells were eliminated and the mass population of puromycin‐resistant cells expressing the shRNAs were obtained by treatment of cells with puromycin (2 μg/mL; Gibco) after infection (24 hours) and continued for one week. Then Western blotting was done to assess the knockdown efficiency of FDPS.

### 2.6 Western blotting

Western blotting was performed as previously research.^26^, ^27^ Briefly, from the 10 cm plates, cell lysates were collected using a buffer for total protein extraction (Beyotime, Shanghai, China) in presence of inhibitors of proteinase and phosphatase (EDTA, 1 mmol/L; sodium pyrophosphate, 10 mmol/L; sodium orthovanadate, 1 mmol/L; NaF, 100 mmol/L; aprotinin, 10 units/mL; leupeptin, 10 mg/mL; PMSF, 1 mmol/L) and BCA Protein Assay Kit from Pierce Biotechnology (Rockford, IL, USA) was used to determine protein concentration. SDS‐PAGE was done to separate the proteins which were then transferred to PVDF membranes supplied by Millipore (Burlington, MA, USA). The blocking of non‐specific binding was done for 1 hour by 5% non‐fat dried milk in TBST (Tris‐buffered saline with Tween‐20) at room temperature. Then overnight probing of membrane with primary antibodies was done at 4°C. After giving three washes in TBST, secondary antibodies specific for species (Thermo Fisher Scientific, Waltham, MA, USA) were added to the membranes, incubated and the detection was done on the imaging system Odyssey from LI‐COR Biosciences (Lincoln, NE, USA). The primary antibodies used are list as follows: FDPS (Invitrogen, #PA5‐28228), claudin‐1 (Invitrogen, #51‐9000), N‐cadherin (Invitrogen, #33‐3900), vimentin (Invitrogen, #PA1‐10003), E‐cadherin (Cell Signaling Technology (Danvers, MA, USA), #3195), cyclin D1 (Cell Signaling Technology, #2978), c‐Myc (Cell Signaling Technology, #5605), β‐catenin (Cell Signaling Technology, #8480), p‐β‐catenin (Cell Signaling Technology, #4176), β‐actin (Sigma‐Aldrich, A5441).

### 2.7 Real‐time PCR

Real‐time PCR was performed as previously studies.^28^, ^29^ Briefly, cells were given one PBS wash, and using Trizol reagent (Takara, Japan), total RNA was extracted and reversely transcribed using RT‐PCR kit PrimeScript from Takara (Japan) as per instructions of the manufacturer. SYBR Premix Ex Taq from Takara (Japan) was used for real‐time quantitative PCR analysis on a 7500 Real‐time PCR system from Applied Biosystems (USA) at the following recommended settings of the thermal cycler: one initial cycle at 95°C for 10 minutes; 40 cycles of 5 seconds at 95°C and 30 seconds at 60°C. Then, the 2^−ΔΔ^ *^Ct^* method was used to determine the relative mRNA expression and the values were normalized to mRNA levels of β‐actin. Primers are list in Table 1.

**TABLE 1:** Primers sequence

	Forward	Reverse
FDPS	5′‐GTGCTGACTGAGGATGAGATG‐3′	5′‐GCTCGATCAGGTTCAGGTAATAG‐3′
CSF‐1	5′‐ATGACAGACAGGTGGAACTGCCAG‐3′	5′‐TCACACAACTTCAGTAGGTTCAGG‐3′
TGF‐β	5′‐CCCAGCATCTGCAAAGCTC‐3′	5′‐GTCAATGTACAGCTGCCGCA‐3′
IL‐4	5′‐TCGGCATTTTGAACGAGGTC‐3′	5′‐GAAAAGCCCGAAAGAGTCTC‐3′
IL‐6	5′‐CCAGCTATGAACTCCTTCTC‐3′	5′‐GCTTGTTCCTCACATCTCTC‐3′
IL‐8	5′‐GTGCAGTTTTGCCAAGGAGT‐3′	5′‐TTATGAATTCTCAGCCCTCTTC‐3′
IL‐13	5′‐GAGTGTGTTTGTCACCGTTG‐3′	5′‐TACTCGTTGGTCGAGAGCTG‐3′
VEGF	5′‐TGCAGATTATGCGGATCAAACC‐3′	5′‐TGCATTCACATTTGTTGTGCTGTAG‐3′
CCL2	5′‐AGAATCACCAGCAGCAAGTGTCC‐3′	5′‐TCCTGAACCCACTTCTGCTTGG‐3′
CCL20	5′‐CTGGCTGCTTTGATGTCAGT‐3′	5′‐CGTGTGAAGCCCACAATAAA‐3′
CCL24	5′‐TAGAGGGCTCTTGGTCACA‐3′	5′‐GTCCTCCAGGTCCATTCATTAC‐3′
CXCL2	5′‐TCCTCAATGCTGTACTGGTCC‐3′	5′‐ATGTTCTTCCTTTCCAGGTC‐3′
CXCL5	5′‐CTCAGTCATAGCCGCAACCGAGC‐3′	5′‐CGCTTCTTTCCACTGCGAGTGC‐3′
MIF	5′‐CTCTCCGAGCTCACCCAGCAG‐3′	5′‐CGCGTTCATGTCGTAATAGTT‐3′
HGF	5′‐GCACCGTCAAGGCTGAGAAC‐3′	5′‐ATGGTGGTGAAGACGCCAGT‐3′
cyclin D1	5′‐CTTCCTCTCCAAAATGCCAG‐3′	5′‐AGAGATGGAAGGGGGAAAGA‐3′
MMP7	5′‐AAACTCCCGCGTCATAGAAAT‐3′	5′‐TCCCTAGACTGCTACCATCCG‐3′
c‐Myc	5′‐TGAGGAGACACCGCCCAC‐3′	5′‐CAACATCGATTTCTTCCTCATCTTC‐3′
β‐catenin	5′‐ACAAACTGTTTTGAAAATCCA‐3′	5′‐CGAGTCATTGCATACTGTCC‐3′
β‐actin	5′‐TGGAATCCTGTGGCATCCATGAAAC‐3′	5′‐AAAACGCAGCTCAGTAACAGTCCG‐3′

### 2.8 Clone formation assay

In each 6‐well plate, suspension of single‐cell was plated at 1000 cells density. Every three days, the replacement of the culture medium was done. After 14 days, fixation and staining of clones were done with 0.1% crystal violet/40% methanol and colonies with more than fifty cells were enumerated under a microscope.

### 2.9 Macrophage depletion assay

The mice were kept under specific conditions that were pathogen‐free as per institutional guidelines. The Ethics Committee of The Third Hospital of Jilin University approved of the animal studies and procedures. Liposomes (PBS and Clodronate) were procured from Sigma. Briefly, intraperitoneal injection of clodronate liposomes (1.4 mg/20 g bodyweight) or PBS liposomes (an equivalent volume) were given to BALB/c mice two times per week until 5th week. At week 2, C57BL/6 mice were injected with 5 × 10^5^ ov‐FDPS or ov‐vector GL261 cells.

### 2.10 ELISA analysis of CCL20

Cells were given one PBS wash and incubated with medium without FBS for 24 hours. Supernatants of cells were collected, centrifuged and immediately used. The normalization of supernatant amount to cell number was done. The CCL20 ELISA kits (Sigma) were used as per instructions of the manufacturer.

### 2.11 Tumour mouse model

The Jackson Laboratory supplied the Female NSG mice (4 to 6‐weeks old) which were then kept in a specific environment free of pathogens. The Animal Care Committee of The Third Hospital of Jilin University approved of the protocols and all processes were carried out as per the institutional guidelines. Subcutaneous inoculation of 5 × 10^5^ cells (n = 6/group) in 100 μL PBS was done into the nude mice. Vernier calipers were used to determine tumour size and the tumour volume of tumour was calculated as per the formula: length × (width)^2^ × 1/2. After 19 days, mice were checked for tumour formation.

### 2.12 Statistical analysis

Graphpad prism 5 software was used for all analyses (*P* < 0.05 was statistically significant). For the mean values of the two groups, the statistical significance was assessed using the two‐tailed unpaired *t* test.

## 3 RESULTS

### 3.1 FDPS is highly expressed in glioma tissues

**FIGURE 1:**
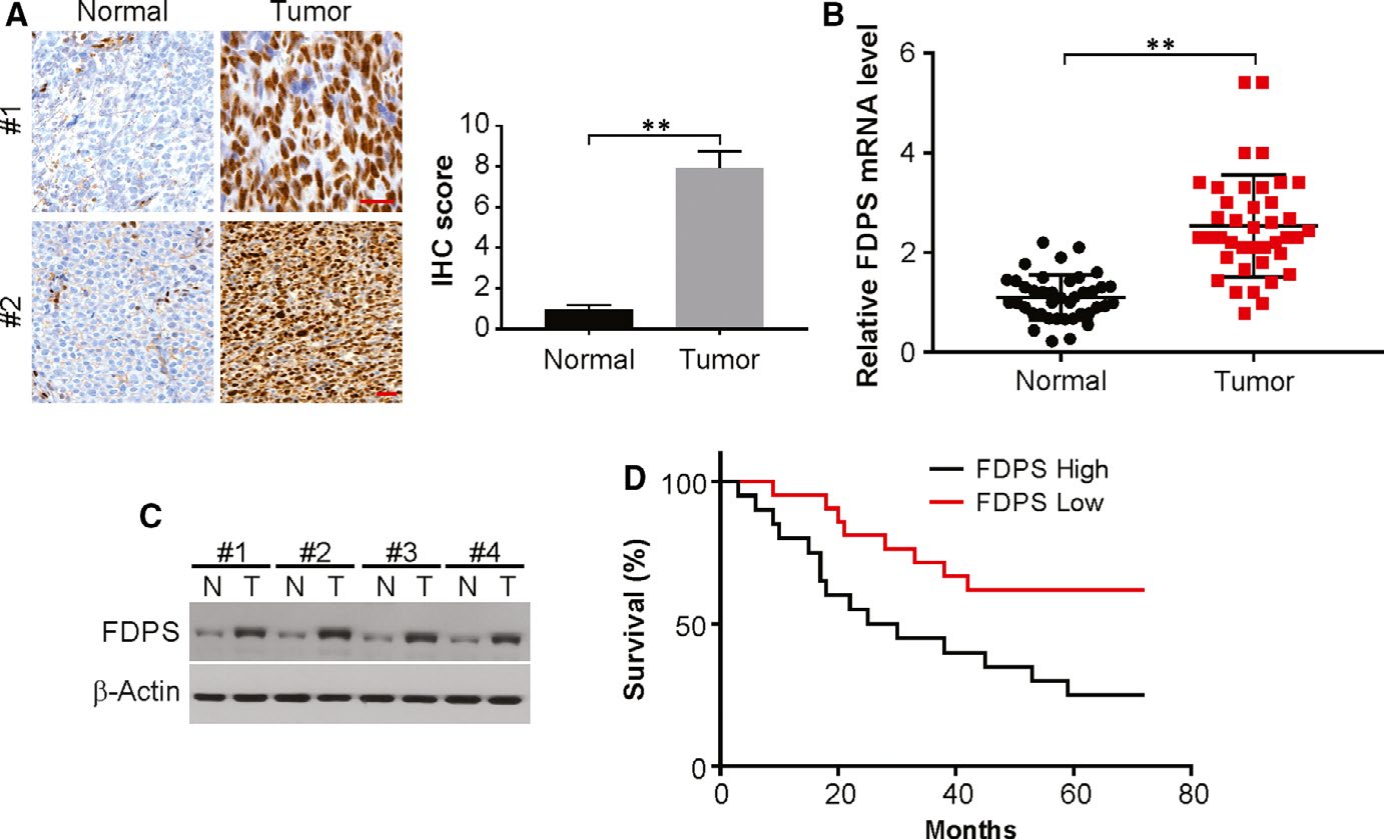
FDPS is expressed at high levels in glioma tissues. The expression of FDPS in glioma tissue and adjacent non‐tumour tissue from same patient was assessed by immunohistochemistry (A), real‐time PCR (B) and Western blotting (C). (D) Kaplan‐Meier curves showing the overall survival of glioma patients with low versus high FDPS expression (*P* = 0.025). Error bars represent the SD, **P < 0.01 (Scale bars, 50 μm)

The expression levels of FDPS were analysed in 40 paraffin‐embedded archived glioma tissues using immunohistochemistry (IHC). The results showed that FDPS levels were elevated in tumour tissues compared with the corresponding adjacent non‐tumour tissues, with strong staining of FDPS detected in 60% of the glioma tissues (Figure 1A). The FDPS mRNA and protein levels were higher than those of corresponding adjacent non‐tumour samples by real‐time PCR and Western blotting (Figure 1B,C). We next evaluated the association of the increased FDPS expression with clinicopathologic features in 40 glioma samples with IHC data. Kaplan‐Meier survival analysis showed that patients with glioma and overexpression of FDPS in their tumours exhibited decreased overall survival compared with those with low expression of FDPS in their tumours (Figure 1D). Taken together, these results suggest that overexpression of FDPS was frequently detected in glioma.

### 3.2 Glioma cell growth is regulated by FDPS

**FIGURE 2:**
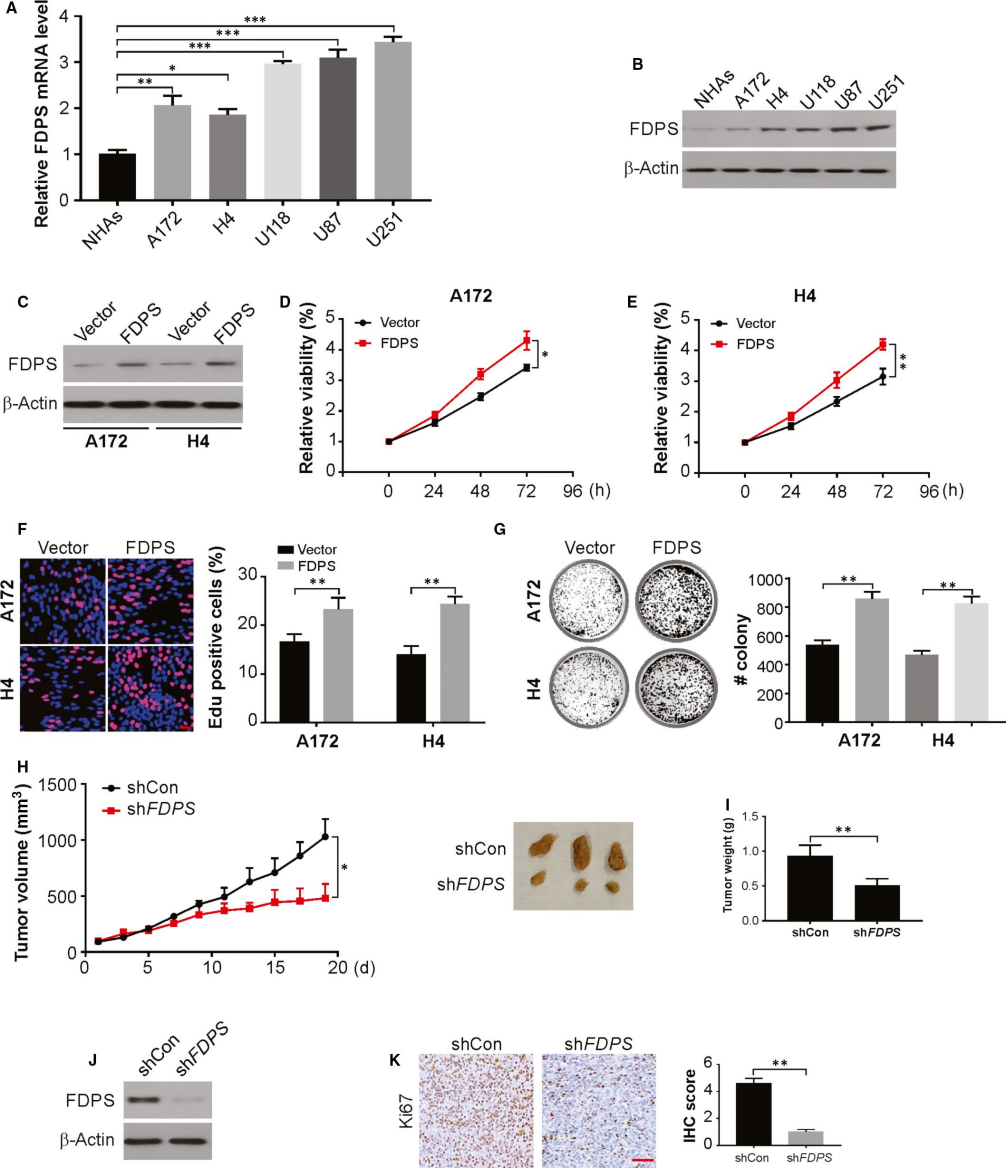
FDPS regulates glioma cell growth. A, The protein expression levels of FDPS in human NHAs and glioma cell lines determined by real‐time PCR. B, The protein expression levels of FDPS in human NHAs and glioma cell lines determined by Western blotting. C, A172 and H4 cells with FDPS overexpression were established. The level of FDPS in these established cell lines was verified by Western blotting. D and E, Cell proliferation was examined by MTS in A172 and H4 cells with FDPS overexpression. F, EdU assay of indicated cells with FDPS overexpression. G, Colony formation assays of indicated cells with FDPS overexpression. H, U87 cells with stable expression of shCon/shFDPS were subcutaneous injected into NSG mice. Tumour size was measured every 2 days. I, The weight of tumours formed at day 17th. J, The FDPS expression in tumours was analysed by Western blotting. K, The expression of Ki67 was evaluated by IHC staining. Error bars represent the SD, *P < 0.05; **P < 0.01; ***P < 0.001 (Scale bars, 50 μm)

We next assessed the oncogenic role of FDPS in glioma, by carrying out Western blotting to examine the expression of FDPS in NHAs cells and in other glioma cell lines (H4, U87, U118, U251 and A172). The FDPS was observed in much lower levels in NHAs cells compared to that in glioma cell lines (Figure 2A,B). When the five glioma cell lines were compared, the highest FDPS expression was seen in U251 and U87 cells, while the lowest level was observed in A172 and H4 cells. Hence, we chose A172 and H4 cells for overexpression of FDPS and U251 and U87 cells for the knockdown of FDPS. Then, FDPS overexpression in A172 and H4 cells and FDPS knockdown in U251 and U87 cells were established. FDPS protein levels in glioma cells were confirmed through Western blotting (Figure 2C; Figure S1A). The knockdown of FDPS lowered cell proliferation to little extent (Figure S2B,C) and caused a decline in EdU‐positive cell percentage (Figure S2D), while overexpression of FDPS facilitated the glioma cell growth (Figure 2D‐F). Likewise, silencing FDPS displayed fewer and smaller colonies in clonogenic assay, whereas overexpression of FDPS augmented clonogenicity (Figure 2G; Figure S2E).

We further examined the association between FDPS and growth of tumour in a xenograft model to extrapolate the in vitro outcomes. Figure 2H,I shows decreased proliferative capacity at the site of implantation of the tumours from U87‐sh*FDPS* cells than controls. Finally, the average tumour weight and volume decreased significantly in sh*FDPS* group (Figure 2H,I). Down‐regulation of FDPS was confirmed by Western blotting in the sh*FDPS* tumours (Figure 2J). Moreover, the tumour cell Ki‐67 percentage score in sh*FDPS* group decreased relatively in comparison to that in shcon group (Figure 2K). Thus, these results indicate that FDPS regulates glioma proliferation.

### 3.3 FDPS promotes glioma migration and invasion in vitro

**FIGURE 3:**
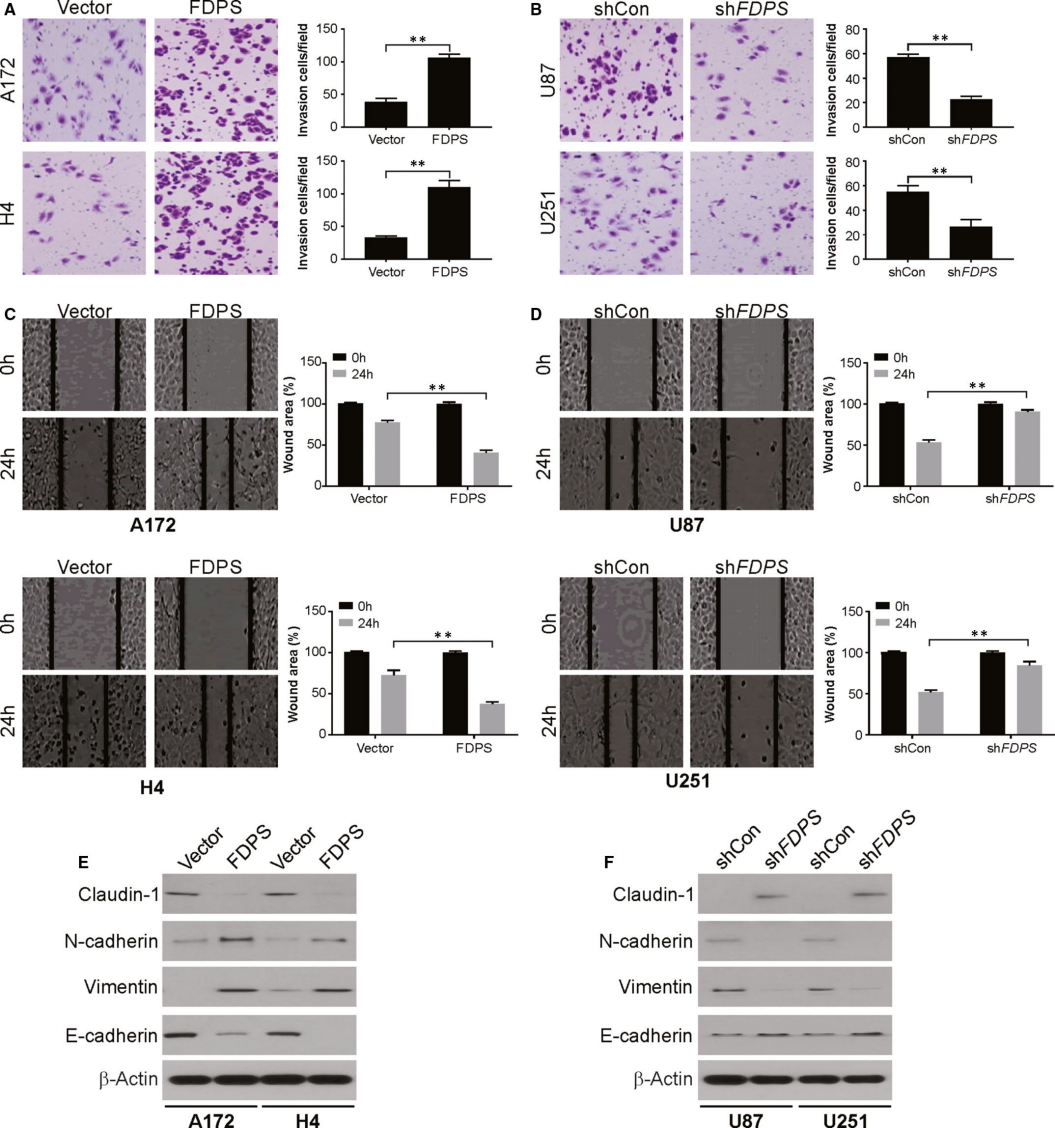
FDPS promotes glioma invasion and migration in vitro. A, The invasion capacity of FDPS in A172 and H4 cells with FDPS overexpression was examined by Matrigel invasion assay. B, The invasion capacity of FDPS in U87 and U251 cells with FDPS silencing was examined by Matrigel invasion assay. C, The migration capacity of FDPS in A172 and H4 cells with FDPS overexpression was examined by wound healing assay. D, The migration capacity of FDPS in U87 and U251 cells with FDPS silencing was examined by wound healing assay. E, The indicated protein level was analysed by Western blotting in A172 and H4 cells with FDPS overexpression. F, The indicated protein level was analysed by Western blotting in U87 and U251 cells with FDPS silencing. Error bars represent the SD, **P < 0.01

Next, we investigated the activity of FDPS in promoting migration and invasion in vitro. The transwell and wound healing assays showed that FDPS promoted invasion and migratory capability in A172 and H4 cells (Figure 3A,C). While, knockdown FDPS suppressed migration and invasion in glioma U87 and U251 cells (Figure 3B,D).

The role of EMT (epithelial‐mesenchymal transition) in facilitating cancer cells with invasive and metastatic properties is well‐document, in addition to its vital part in progression of tumours. In this study, we found knockdown of FDPS enhanced the expression of epithelial markers (claudin‐1 and E‐cadherin), but repressed levels of mesenchymal markers (vimentin and N‐cadherin) in U87 and cells. Contrarily, overexpression of FDPS had an opposite effect on A172 and H4 cells (Figure 3E,F). Thus, FDPS exerts a significant role in glioma invasion and migration.

### 3.4 FDPS promotes tumour growth in a macrophage‐dependent manner

**FIGURE 4:**
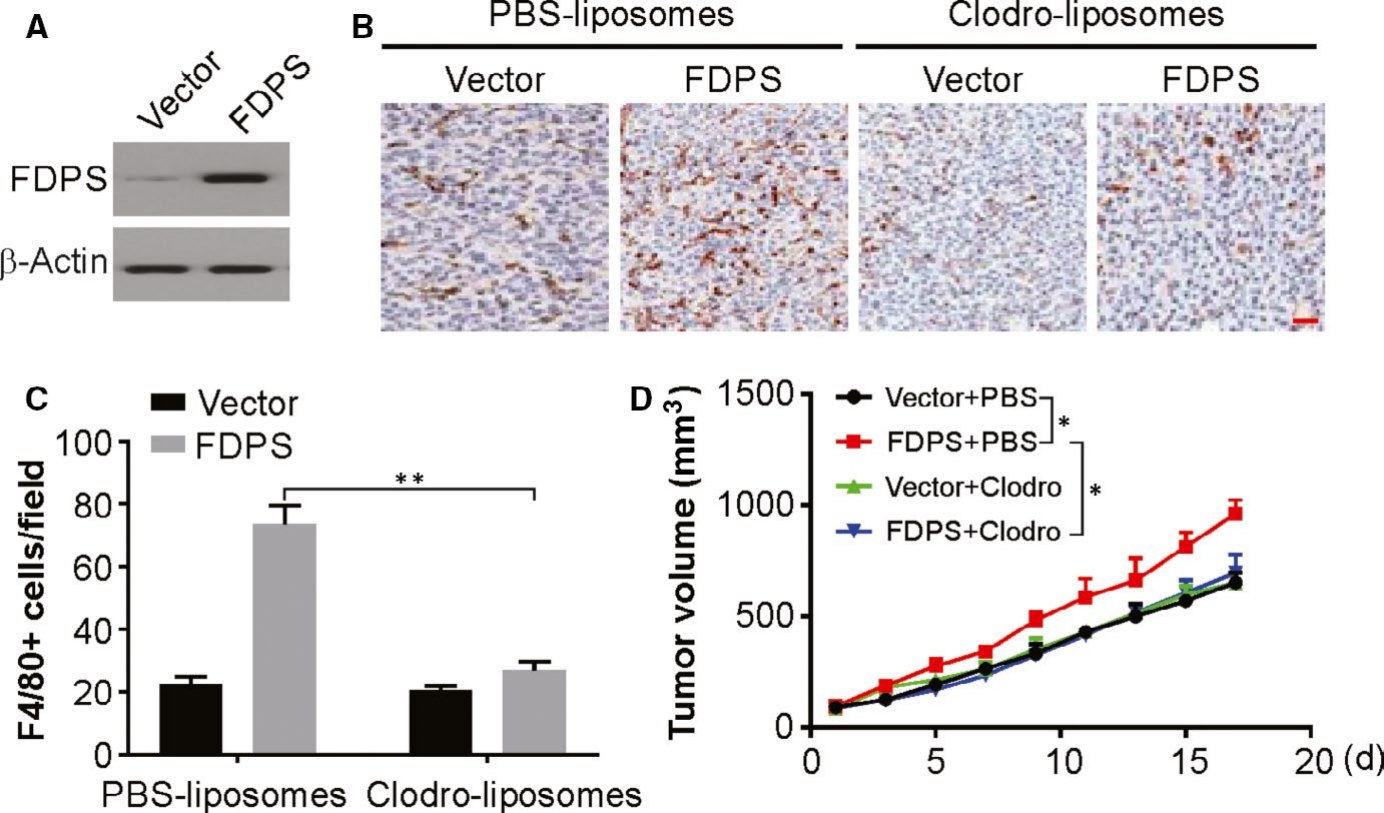
FDPS promotes tumour growth in a macrophage‐dependent manner. A, The level of FDPS in GL261 cells was analysed by Western blotting. B and C, Representative immunohistochemistry of F4/80+ cells in sections from glioma tumours obtained from C57BL/6J mice treated with clodronate liposomes or PBS liposomes. D, Volume of GL261 tumours treated as indicated. Error bars represent the SD, *P < 0.05; **P < 0.01 (Scale bars, 50 μm)

To demonstrate whether the oncogenic roles of FDPS in glioma are macrophage‐dependent, we investigated the susceptibility of macrophages to liposomal clodronate treatment. To address this issue, we overexpressed FDPS in a mouse glioma cell line GL261, which showed faint FDPS protein expression. The overexpression efficiency is shown in Figure 4A. Next, we performed syngeneic mouse model with FDPS‐overexpressing and control cell lines injected in the C57BL/6 mice. For 2 weeks, the mice were given clodronate liposomes or phosphate‐buffered saline (PBS) liposomes pre‐treatment and additional three weeks treatment following tumour cell injection. The clodronate liposomal treatment was adequate to decrease the number of F4/80^+^ macrophages in tumour of C57BL/6 mice (Figure 4B,C). Meanwhile, we also noticed more macrophage infiltration in FDPS‐overexpressing group in comparison to the control group (Figure 4B,C). Our findings also revealed that increased tumour burden induced by FDPS‐overexpressing was largely attenuated in mice that underwent liposomal clodronate treatment (Figure 4D), suggesting that the tumour‐promoting effects of FDPS in glioma, at least in part, are mediated by macrophage infiltration in the tumour microenvironment.

### 3.5 CCL20 mediate the promoting role of FDPS in macrophage infiltration

**FIGURE 5:**
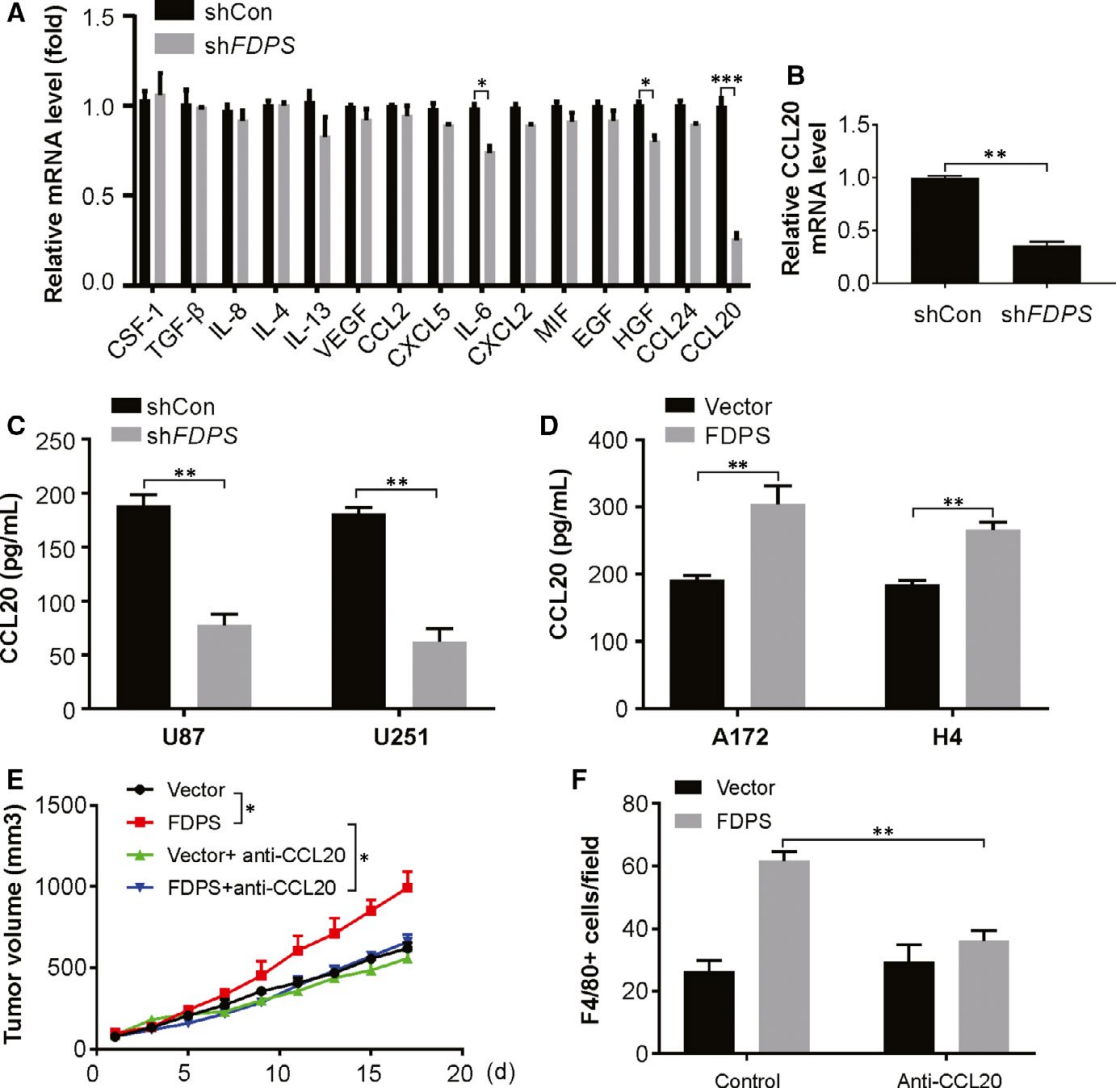
CCL20 mediates the promoting role of macrophage infiltration induced by FDPS. A, The mRNA level of indicated chemokines in the conditional medium from U87 cells with or without FDPS silencing was analysed by real‐time PCR. B, The mRNA level of CCL20 in the conditional medium from U251 cells with or without FDPS silencing was analysed by real‐time PCR. C, The level of CCL20 in the conditional medium from U87 and U251 cells with or without FDPS silencing was analysed by ELISA. D, The level of CCL20 in the conditional medium from A172 and H4 cells with or without FDPS overexpression was analysed by ELISA. E, Volume of parental and FDPS‐overexpressing GL261 tumours treated as indicated. F, F4/80+ cells in sections from indicated glioma tumours. Error bars represent the SD, *P < 0.05; **P < 0.01; ***P < 0.001

To identify the chemokines regulated by FDPS that promote macrophage infiltration, the chemokine profiles of CM from shcon and sh*FDPS* U87 cells were analysed using real‐time PCR. CCL20 was significantly decreased in the CM of sh*FDPS* cells compared with shcon cells (Figure 5A). Real‐time PCR showed that FDPS knockdown drastically inhibited the mRNA level of CCL20 in U251 cells (Figure 5B). ELISA assay further confirmed the reduction of CCL20 in the CM of sh*FDPS* cells and FDFS overexpression cells (Figure 5C,D). To further investigate the role of CCL20 in FDPS‐mediated macrophage infiltration, we treated the mice bearing control or FDPS‐overexpressing tumour with CCL20 neutralizing antibody. As shown in Figure 5E,F, CCL20 neutralizing antibody treatment decreased FDPS‐induced tumour growth and macrophage infiltration. Together, we conclude that CCL20 is required for FDPS‐mediated macrophage infiltration in glioma.

### 3.6 FDPS enhances Wnt/β‐catenin signalling

**FIGURE 6:**
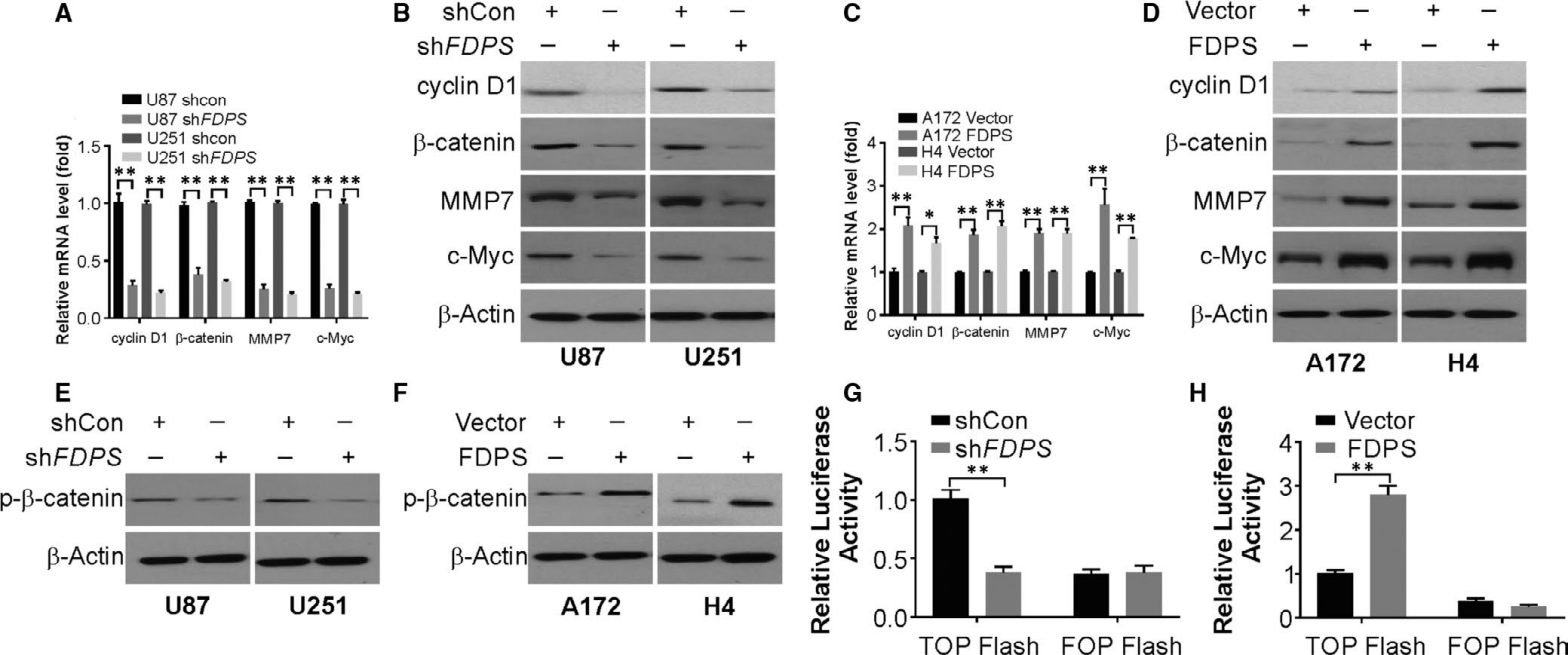
FDPS regulates Wnt/β‐catenin signalling pathway activation. (A) mRNA level of indicated genes in U87 and U251 cells with or without FDPS silencing was analysed by real‐time PCR. (B) Protein level of indicated genes in U87 and U251 cells with or without FDPS silencing was analysed by Western blotting. (C) mRNA level of indicated genes in A174 and H4 cells with or without FDPS overexpression was analysed by real‐time PCR. (D) Protein level of indicated genes in A172 and H4 cells with or without FDPS overexpression was analysed by Western blotting. (E) Protein level of p‐β‐catenin in U87 and U251 cells with or without FDPS silencing was analysed by Western blotting. (F) Protein level of p‐β‐catenin in A172 and H4 cells with or without FDPS overexpression was analysed by Western blotting. The activity of TCF/β‐catenin reporter (TOP/FOP Flash) in FDPS knockdown (G) and FDPS‐overexpressing cells (H). Error bars represent the SD, *P < 0.05; **P < 0.01

Next, we observed that *FDPS* knockdown in U87 cells remarkably lowered transcript and protein levels of the components of Wnt/β‐catenin signalling pathway, such as cyclin D1, β‐catenin, MMP7 and c‐Myc (Figure 6A,B). However, exogenous FDPS expression elevated transcript and protein levels of cyclin D1, β‐catenin, MMP7 and c‐Myc (Figure 6C,D). These findings support that FDPS activates the Wnt/β‐catenin signalling pathway, and FDPS knockdown suppresses the signalling pathway of Wnt/β‐catenin. For more robust argument, we evaluated the level of the which mediates target gene activation, the active form of β‐catenin and observed significantly decrease in activated β‐catenin when FDPS was knocked down and elevated when FDPS in glioma cells, where it was ectopically expressed (Figure 6E,F). The effect of FDPS on signalling activity of β‐catenin was also estimated via the TOP/FOP Flash assay, a well‐established reporter assay of dual‐luciferase TCF/β‐catenin. The TOP Flash reporter contains TCF‐responsive sites, whereas the FOP Flash reporter, is a negative control, and harbours mutated TCF binding sites. When we depleted FDPS, TOP Flash luciferase activity declined significantly, while exogenous FDPS enhanced the TCF reporter transactivation (Figure 6G,H). Thus, FDPS plays a significant role as a positive regulator of oncogenic function of Wnt/β‐catenin.

### 3.7 Wnt/β‐catenin pathway is responsible for CCL20 expression

**FIGURE 7:**
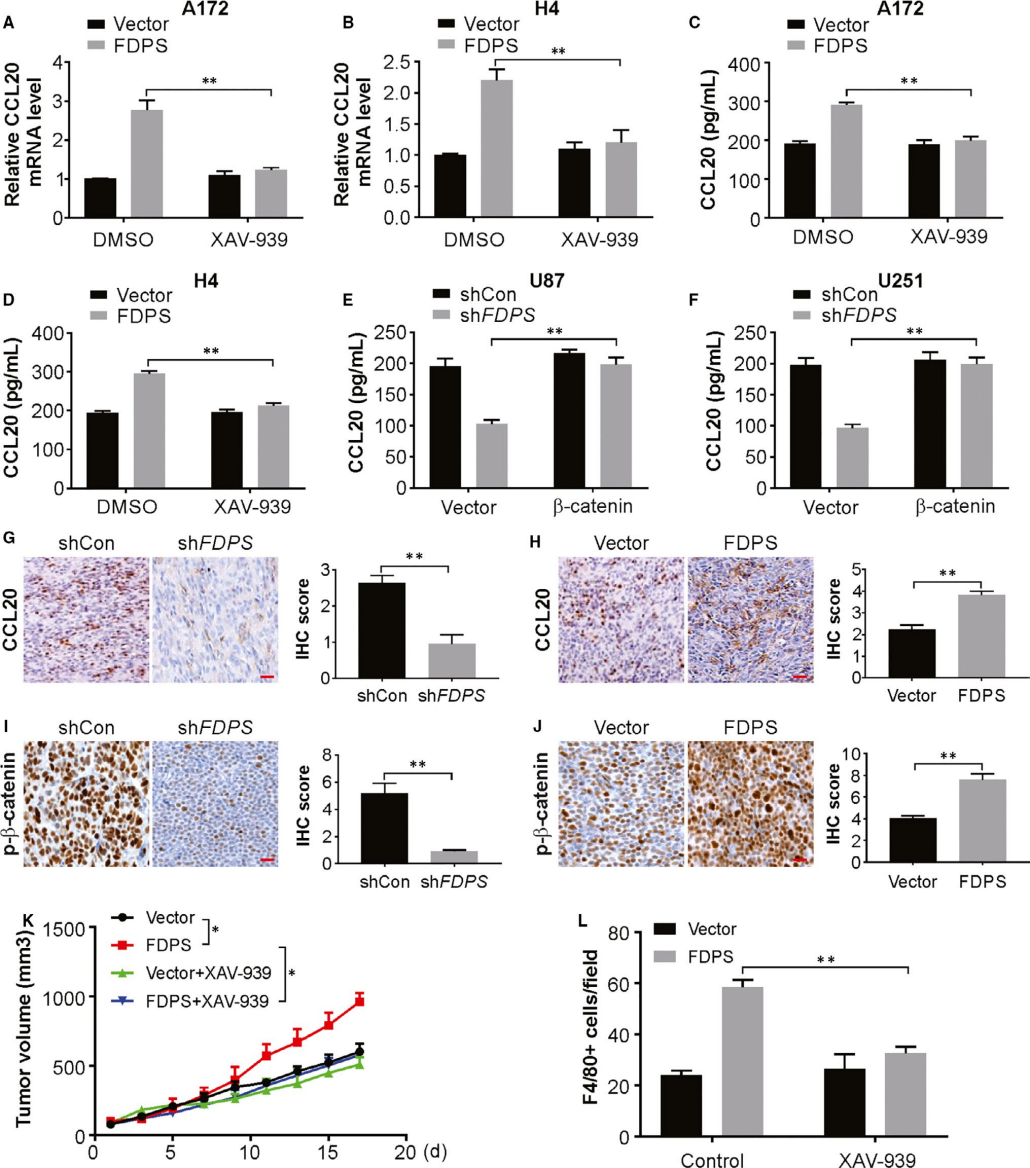
Wnt/β‐catenin signalling pathway is required for FDPS‐mediated CCL20 induction. A, mRNA level of CCL20 in the conditional medium from A172 FDPS‐overexpressing cells with or without XAV‐939 pre‐treatment was analysed by real‐time PCR. B, mRNA level of CCL20 in the conditional medium from H4 FDPS‐overexpressing cells with or without XAV‐939 pre‐treatment was analysed by real‐time PCR. C, The level of CCL20 in the conditional medium from A172 FDPS‐overexpressing cells with or without XAV‐939 pre‐treatment was analysed by ELISA. D, The level of CCL20 in the conditional medium from H4 FDPS‐overexpressing cells with or without XAV‐939 pre‐treatment was analysed by ELISA. E, The level of CCL20 in the conditional medium from U87 FDPS knockdown cells with or without β‐catenin overexpression was analysed by ELISA. F, The level of CCL20 in the conditional medium from U251 FDPS knockdown cells with or without β‐catenin overexpression was analysed by ELISA. G, CCL20 was analysed by IHC in the shcon and sh*FDPS* U87 tumours. H, CCL20 was analysed by IHC in the vector and FDPS GL261 tumours. I, p‐β‐catenin was analysed by IHC in the shcon and sh*FDPS* U87 tumours. J, p‐β‐catenin was analysed by IHC in the vector and FDPS GL261 tumours. K, Volume of parental and FDPS‐overexpressing GL261 tumours treated as indicated. L, F4/80+ cells in sections from indicated glioma tumours. Error bars represent the SD, *P < 0.05; **P < 0.01

Next, to evaluate the dependence of Wnt/β‐catenin in chemokine production, A172 and H4 cells were treated with the small‐molecule Wnt/β‐catenin inhibitor XAV‐939. As expected, XAV‐939 led to Wnt/β‐catenin suppression and significantly reduced the transcript and protein level of CCL20 in both two cell lines (Figure 7A‐D). Additionally, ectopic expression of constitutively active β‐catenin largely attenuated the decreased CCL20 level induced by FDPS inhibition (Figure 7E,F).

To decipher the FDPS‐Wnt/β‐catenin‐CCL20 axis in vivo, we performed immunohistochemistry analysis in the tumour tissues from syngeneic mouse model. As a result, we found that FDPS knockdown significantly reduced CCL20 protein expression in subcutaneous xenograft model, while overexpression of FDPS remarkably promoted CCL20 protein levels in syngeneic tumour tissues (Figure 7G,H). At the same time, low p‐β‐catenin level was found in FDPS knockdown xenograft tumours, as well as, overexpression of FDPS increased the p‐β‐catenin expression in syngeneic tumours (Figure 7I,J). Moreover, XAV‐939 treatment reduced FDPS‐mediated tumour growth and macrophage infiltration (Figure 7K,L). Therefore, Wnt/β‐catenin activation is necessary for the induction of inflammatory chemokine expression induced by FDPS.

## 4 DISCUSSION

In this study, we found that FDPS is overexpressed in glioma compared with corresponding normal tissues. We also identified FDPS, which regulated the microenvironment of tumour and thus has a significant part in the tumour progression of glioma. FDPS activated CCL20 expression by activating Wnt/β‐catenin signalling pathway. Moreover, FDPS‐induced CCL20 modulated the tumour microenvironment through TAMs infiltration in glioma tissues. Thus, the current observations impart better insight into the translational mechanisms of the modulation of the tumour microenvironment mediated by FDPS that advances the tumour progression of glioma.

As a branch point enzyme, FDPS (Farnesyl Diphosphate Synthase) is involved in sterols and isoprenylated cellular metabolites synthesis.^11^ It exhibits immunoregulatory functions, and its expression and activity have been also studied in human certain other neoplastic disorders and glioma.^9^ Certainly, high levels of FDPS mRNA along with of the transcripts of isoprenoid pathway genes have been associated with poor prognosis and decreased survival rate in a six microarray datasets meta‐analysis of primary breast cancers.^30^ The H‐ras and K‐ras transformed FRTL5 thyroid cells and FDPS, exhibited enhanced FDPS expression in cooperation with H‐RAS oncogene, and exhibited potential for neoplastic transformation in primary embryo fibroblast rat cells.^31^

Researchers indicated that TAMs are one of the M2‐like macrophages because of their high expression of anti‐inflammation marker genes, including IL‐10 (interleukin‐10) and IL‐1Rα (IL‐1 receptor alpha), which contribute highly towards growth of tumour and subsequent development.^32^ The monocytes are recruited by primary TAMs by secreting chemotactic factors CCL2, ‐5, ‐7, as well as CXCL8 and ‐12 which can be polarized to M2‐like phenotype with the stimulation of IL‐4, IL‐6, IL‐10, IL‐13 and TGF‐β (transforming growth factor‐beta).^16^, ^33^, ^34^ Furthermore, tumour‐promoting growth factors from TAMs, like EGF (epidermal growth factor), also facilitate to formation of vascular tissues and modulate immune response.^20^, ^35^ In this process, the synthesis of MMPs (matrix metalloproteinase) which significantly affect angiogenesis is regulated by VEGF (vascular endothelial growth factor), PDGF (platelet derived growth factor), FGF (fibroblast growth factor) and TGF‐β.^36^, ^37^ TAMs have a There is a special period of transition of TAMs from M1‐ to M2‐like phenotype, indicating that they do not just belong to M1‐ or M2‐like phenotype throughout the progression of tumour.^38^ In the initial stage of tumour formation, TAMs exhibit M1‐like and then transform to the M2‐like phenotype.^21^

Next, we uncovered that FDPS is closely associated with macrophage infiltration in glioma. The infiltration of immune cells is, among other events, of great importance for antitumour immune response, and is known to have a strong impact on the outcome of human cancers.^39^ In human cancers macrophages are an important component of the leucocyte infiltrate and play a major role in orchestrating the cancer‐related inflammation.^40^ Tumour‐derived factors stimulated the recruitment and differentiation of macrophages.^41^ In this study, FDPS expression correlates with the majority of gene signatures related to macrophages and are closely associated with macrophage density, suggesting the tumour‐promoting effects of FDPS in glioma might be linked to the macrophage infiltration. There is a strong association between increased macrophage density and poor prognosis. Macrophages can suppress Th1 cell and antitumour cytotoxic T lymphocyte responses, contribute to matrix remodelling and facilitating tumour cell migration and invasion and promote tumour angiogenesis and growth.^42^ In line with this, we showed that deletion of macrophages by liposomal clodronate largely compromises the growth‐promoting effects of FDPS in syngeneic model, suggesting that the oncogenic roles of FDPS in glioma are largely dependent on macrophages.

In conclusion, our study showed that dysregulated neurotransmitter receptor FDPS promotes cancer aggressiveness and correlates macrophage infiltration in glioma and provides a new insight into the roles of neurotransmitter receptor in immune modulation. These findings above demonstrate that point towards the FDPS/Wnt/β‐catenin/CCL20/macrophage axis as potential therapeutic target in glioma.

## CONFLICT OF INTEREST

The authors declare no conflict of interest.

## AUTHOR CONTRIBUTION

**Zhuo Chen:** Investigation (equal); Project administration (equal). **Guangyong Chen:** Data curation (equal); Investigation (equal). **hang zhao:** Conceptualization (equal); Investigation (equal); Project administration (equal).

## Supporting information

Supplementary Material

## Data Availability

The data that support the findings of this study are available from the corresponding author upon reasonable request.
