# Supplementary material for: FDPS promotes glioma growth and macrophage recruitment by regulating CCL20 via Wnt/β‐catenin signalling pathway
Source: J Cell Mol Med. 2020 Jun 28;24(16):9055–66. doi: 10.1111/jcmm.15542 (PMC7417684; doi:10.1111/jcmm.15542)
Supplement: Supplementary file 1 — Supplementary Material [file JCMM-24-9055-s001.docx]

**FDPS promotes glioma growth and macrophage recruitment by regulating CCL20 via Wnt/β-catenin signaling pathway**

Zhuo Chen, Guangyong Chen, Hang Zhao*

Neurosurgery department, The Third Hospital of Jilin University, Changchun, Jilin 130033, China.

*Corresponding author

Hang Zhao

Neurosurgery department, The Third Hospital of Jilin University, NO. 126, Xiantai Street, Changchun, Jilin, 130033, China. Email: [zhaohang@jlu.edu.cn](mailto:zhaohang@jlu.edu.cn)

**Running title:** FDPS recruits TAMs in glioma


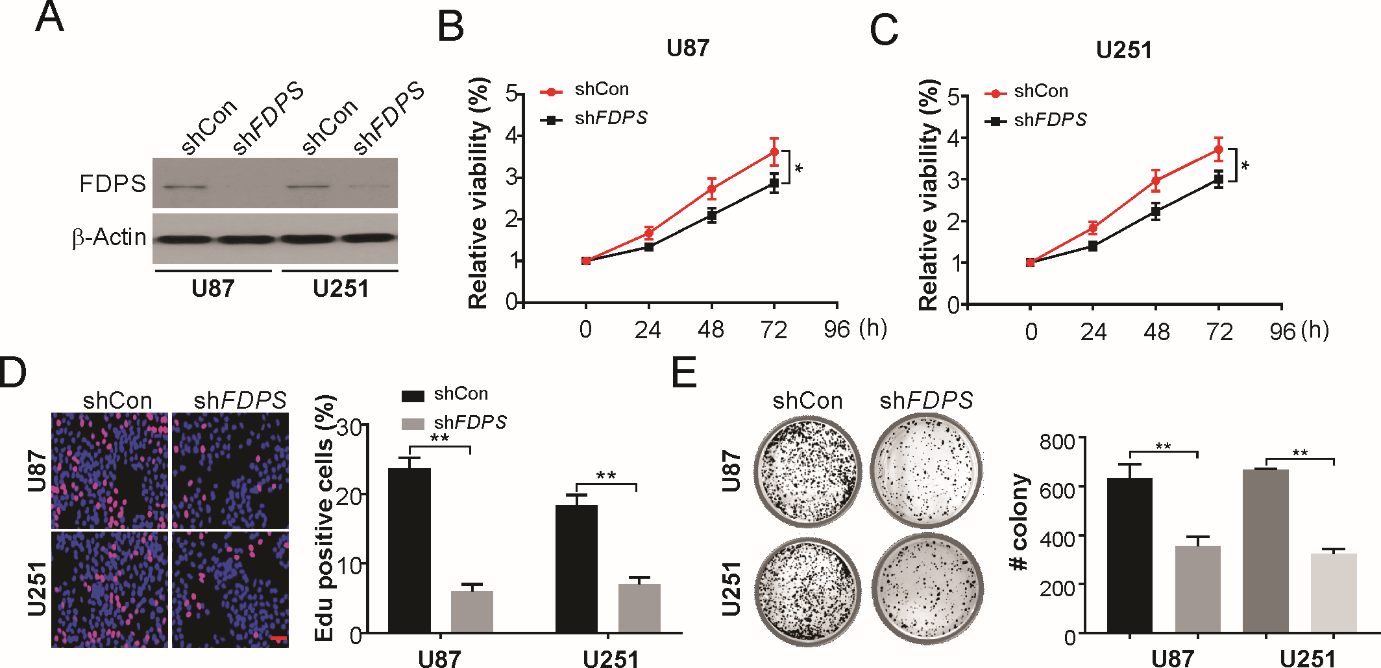


**Figure S1. FDPS regulates glioma cell growth. (A)** U87 and U251 cells with silencing FDPS expression were established. The level of FDPS in these established cell lines was veriﬁed by Western blotting. **(B) and (C)** Cell proliferation was examined by MTS in U87 and U251 cells with FDPS silencing. **(D)** EdU assay of indicated cells with FDPS silencing. **(E)** Colony formation assays of indicated cells with FDPS silencing. Error bars represent the SD, ** P< 0.01.


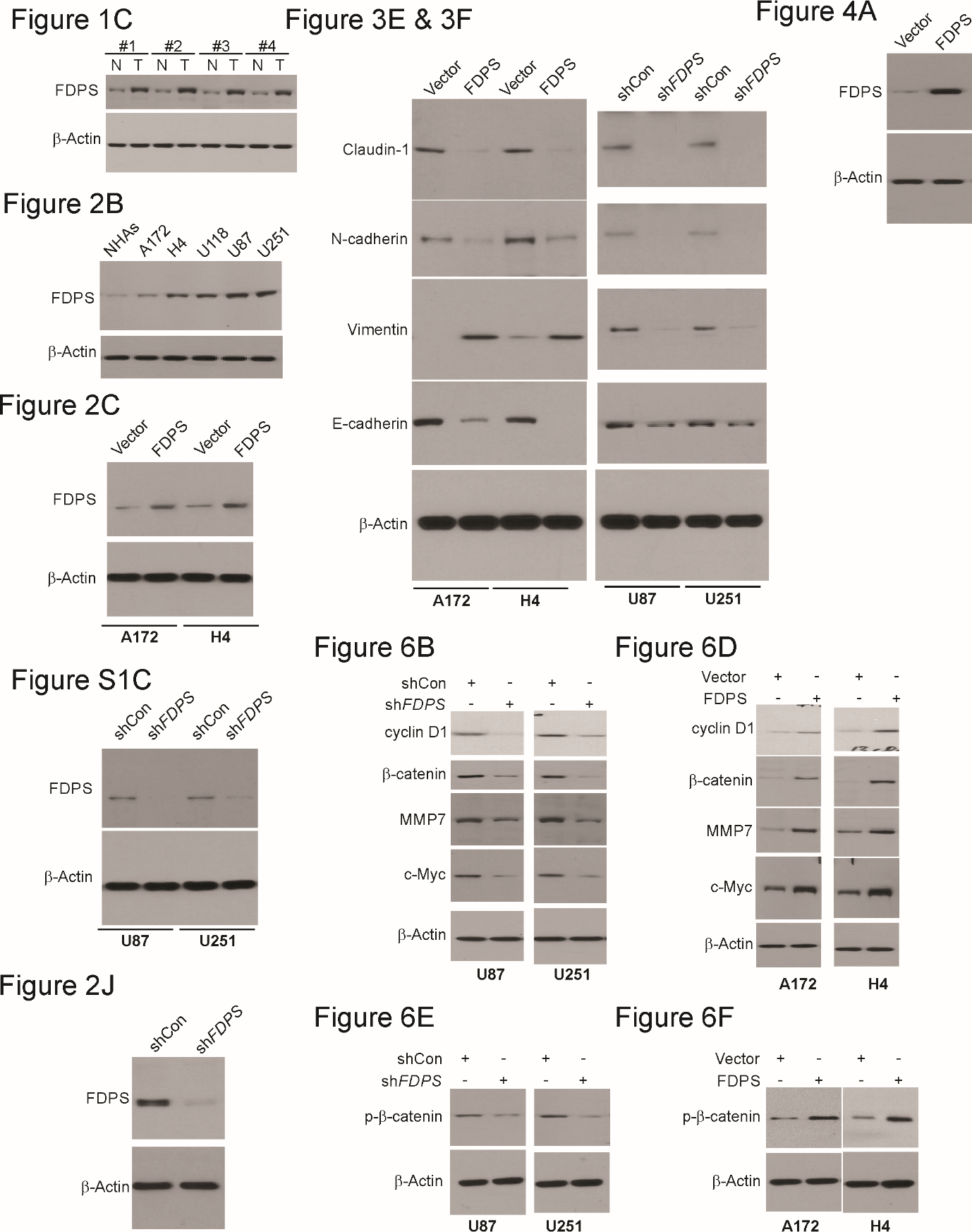


**Figure S2.** Unprocessed western blots.
